# Using the Thickness Map from Macular Ganglion Cell Analysis to Differentiate Retinal Vein Occlusion from Glaucoma

**DOI:** 10.3390/jcm9103294

**Published:** 2020-10-14

**Authors:** Nam Ho Lee, Kee Sup Park, Han Min Lee, Jung Yeul Kim, Chang-sik Kim, Kyoung Nam Kim

**Affiliations:** 1 Mindeulle Eye Clinic, Boeun 28950, Korea; 74amg@naver.com; 2 Department of Ophthalmology, Chungnam National University College of Medicine, Daejeon 35015, Korea; red-mirr@hanmail.net (K.S.P.); lihanil12@naver.com (H.M.L.); kimjy@cnu.ac.kr (J.Y.K.); kcs61@cnu.ac.kr (C.-s.K.)

**Keywords:** primary open-angle glaucoma, retinal vein occlusion, optical coherence tomography, ganglion cell-inner plexiform layer

## Abstract

Purpose: We hypothesized that the thickness map from macular ganglion cell analysis (GCA) acquired from spectral-domain optical coherence tomography can be used to differentiate retinal vein occlusion (RVO) from glaucoma. Methods: In this retrospective case control study, 37 patients with resolved RVO and 74 patients with primary open-angle glaucoma (POAG) were enrolled. Two independent examiners diagnosed patients with RVO or POAG based on the topographic pattern in the GCA thickness map. Inter-observer agreement for a decision between RVO and POAG was assessed using kappa statistics. Diagnostic specificity and accuracy were calculated. Results: Inter-observer agreement was good, with a kappa value of 0.765 (95% confidence interval, 0.634–0.896, *p* < 0.001). The diagnostic specificity of RVO from POAG using the GCA thickness map was 93.2% and diagnosis accuracy was 80.4%. Conclusions: An irregular GCA thickness map represents a simple and convenient differential diagnostic clue to distinguish RVO from POAG.

## 1. Introduction

Retinal vein occlusion (RVO) is the second most frequent vascular disease of the retina [1]. In the acute phase, flame-shaped retinal hemorrhage, hard exudate, and edema are observed, commonly accompanied by a patient’s complaints of an acute reduction in visual acuity with fovea involvement. Clinical diagnosis of RVO is relatively straightforward. However, after resolution of the acute phase, differential diagnosis from other diseases, such as glaucoma, may be difficult in cases without subjective symptoms of deceased visual acuity and objective finding of abnormally tortuous or collateral vessels.

Glaucoma is one of the most common causes of irreversible blindness worldwide. Thinning of the peripapillary retinal nerve fiber layer (RNFL), which is mainly composed of axons of the retinal ganglion cells, provides an important diagnostic clue of glaucoma. However, many other diseases also involve RNFL thinning, such as compressive optic neuropathy, ischemic optic neuropathy, and hypertensive retinopathy [2,3,4,5,6,7,8]. RVO is one of them [9].

Spectral-domain optical coherence tomography (SD-OCT) has become an essential tool for the diagnosis and management of glaucoma, as opposed to an ancillary test. In addition to RNFL analysis in optic disc scans, ganglion cell analysis (GCA) in macular scans is reportedly helpful in the diagnosis of glaucoma [10,11,12,13,14,15,16]. In particular, the thickness map of the GCA yields a holistic topographic pattern of the macular ganglion cell-inner plexiform layer (GCIPL). In glaucoma, the topographic pattern of GCIPL thinning is smooth, without any abrupt changes, and is regularly arcuate, with characteristic distribution of the retinal ganglion cell axons of the RNFL [10,11,12]. This suggests that the GCIPL thinning pattern in RVO may be irregular and/or less arcuate than that associated with glaucoma. If this is the case, the GCA thickness map should help to differentiate RVO and glaucoma. Our speculation is based on the fact that in glaucoma, the RNFL and ganglion cells are the primary sites of damage; in contrast, in RVO, damage in the RNFL and ganglion cells appears secondarily due to retinal vascular occlusion.

In this study, we compared GCA thickness maps between primary open-angle glaucoma (POAG) patients and RVO patients, qualitatively. A quantitative comparison was performed with respect to RNFL thickness, GCIPL thickness, and overall macular thickness between the two diseases.

## 2. Materials and Methods

This retrospective case control study was approved by the Institutional Review Board of Chungnam National University Hospital, which waived the requirement for informed consent from participants (IRB number: 2016-10-020-001). The study was conducted in accordance with the requirements of the Declaration of Helsinki. Patients with RVO and patients with POAG who visited our department were consecutively enrolled from 1 June 2013 to 30 September 2017.

### 2.1. Patients

All participants underwent a thorough ophthalmic examination, including measurement of best-corrected visual acuity (BCVA), auto-refractometry, slit-lamp biomicroscopy, Goldmann applanation tonometry, gonioscopy, dilated fundus examination, fundus photography, and SD-OCT (Cirrus HD OCT; Carl Zeiss Meditec, Dublin, CA, USA). The 24-2 Swedish Interactive Threshold Algorithm standard automated visual field test (Humphrey Visual Field Analyzer; Carl Zeiss Meditec) was performed in patients with POAG.

All participants were required to meet the following criteria for inclusion: BCVA ≥ 20/30 and an axial length ≤ 26.0 mm. Patients with additional retinal or optic nerve disorder or a history of intraocular surgery, with the exception of those having undergone uncomplicated cataract surgery, were excluded.

### 2.2. Retinal Vein Occlusion

The diagnosis of RVO was based on flame-shaped retinal hemorrhages in the distribution of occluded retinal veins, with an apex of the obstructed tributary system. Branch and hemi-central RVO were selected in this study; these two forms of RVO exhibit altitudinal damage in the superior hemisphere or inferior hemisphere, which may be more similar to glaucoma as opposed to central RVO [11,12]. At the time of study recruitment, patients were enrolled if they satisfied all of the following criteria: at least 6 months had elapsed from the acute phase, the retinal hemorrhage and macular edema had subsided, intraocular pressure was below 22 mmHg, and the patient presented with open angles on gonioscopy in both eyes. Recovery of macular edema was defined as the overall macular thickness (measured from the inner limiting membrane to the retinal pigment epithelium) ranging below the 95% distribution of the normative database and with no cystic space on macular cube scans or high-definition SD-OCT raster scans.

### 2.3. Primary Open-Angle Glaucoma

POAG diagnosis was based on glaucomatous optic disc change, reproducible glaucomatous visual field defect on the Humphrey perimetry, and open angles on gonioscopy. Glaucomatous optic disc changes were characterized as focal or diffuse neuroretinal rim thinning, localized notching, or RNFL defects with corresponding visual field defects. Glaucomatous visual field defects were defined if they met two of the following three criteria: the presence of a cluster of three points on a pattern deviation probability plot at *p* < 0.05, one of which was at *p* < 0.01; a pattern standard deviation at *p* < 0.05; or glaucoma hemifield test results outside normal limits [17,18].

One of the eyes in POAG patients was matched with an eye in RVO patients in terms of age (within ±5 years) and average RNFL thickness (within ±5 μm) consecutively enrolled, with a ratio of 2:1.

### 2.4. Spectral-Domain Optical Coherence Tomography

A Cirrus HD OCT system was used in this study. Optic disc cube 200 × 200 scans and macular cube 512 × 128 scans were performed through a full dilated pupil by a single experienced examiner. For inclusion, SD-OCT images acquired from optic disc cube and macular cube scans were required to have a signal strength ≥7, without the presence of artefacts caused by eye motion, blinking, misalignment, or segmentation error.

In optic disc cube scans, RNFL thickness was measured using a 3.46-mm-diameter scan circle centered on the optic disc. Average and quadrant (superior, nasal, inferior, and temporal) RNFL thicknesses were used in the study analysis.

In macular cube scans, the GCA algorithm measured GCIPL thickness within an annulus with inner vertical and horizontal diameters of 1 and 1.2 mm, respectively, and outer vertical and horizontal diameters of 4 and 4.8 mm, respectively. The average, six sectoral (superior, superonasal, inferonasal, inferior, inferotemporal, and superotemporal), and minimum GCIPL thicknesses were used in this study. The thickness map of the GCA was used for qualitative comparison between RVO and POAG. This map provides a topographic pattern of the GCIPL thickness using a continuous color code: with red/white color indicating thickening and blue/black color indicating thinning. Next, overall macular thickness measurements corresponding to the Early Treatment of Diabetic Retinopathy Study (ETDRS) areas were examined. ETDRS areas were defined by three concentric rings (central, inner, and outer circles) centered on the fovea, with diameters of 1, 3, and 6 mm, respectively, and with the inner and outer rings divided into four quadrants. The macular thicknesses of nine areas were analyzed: the central circle, the inner circle quadrant (superior, nasal, inferior, and temporal), and the outer circle quadrant (superior, nasal, inferior, and temporal).

For convenience, we defined ‘damaged hemisphere’ as follows for the two conditions. In patients with RVO, ‘damaged hemisphere’ was defined as a hemisphere with vascular occlusion. In patients with POAG, ‘damaged hemisphere’ was defined as a hemisphere with a thinner RNFL, in which the RNFL thickness in the superior hemisphere was calculated by the sum of the clock hour RNFL thickness from 10 to 2 o/c, and the RNFL thickness in the inferior hemisphere was the sum of the clock hour thickness from 4 to 8 o/c. To compare the RNFL, GCIPL, and macular thickness parameters between RVO and POAG, while avoiding confounding factors caused by differences in prevalence in the superior or inferior hemisphere according to both diseases, SD-OCT results in patients with a ‘damaged hemisphere’ in the superior hemisphere were reversed (converted upside down): specifically, the superior was interchanged with the inferior, superotemporal with inferotemporal, and superonasal with inferonasal. As a result, the ‘damaged hemisphere’ was located inferiorly in all enrolled patients, regardless of RVO or POAG.

### 2.5. Qualitative Analysis of the Thickness Map of the GCA

**Figure 1:**
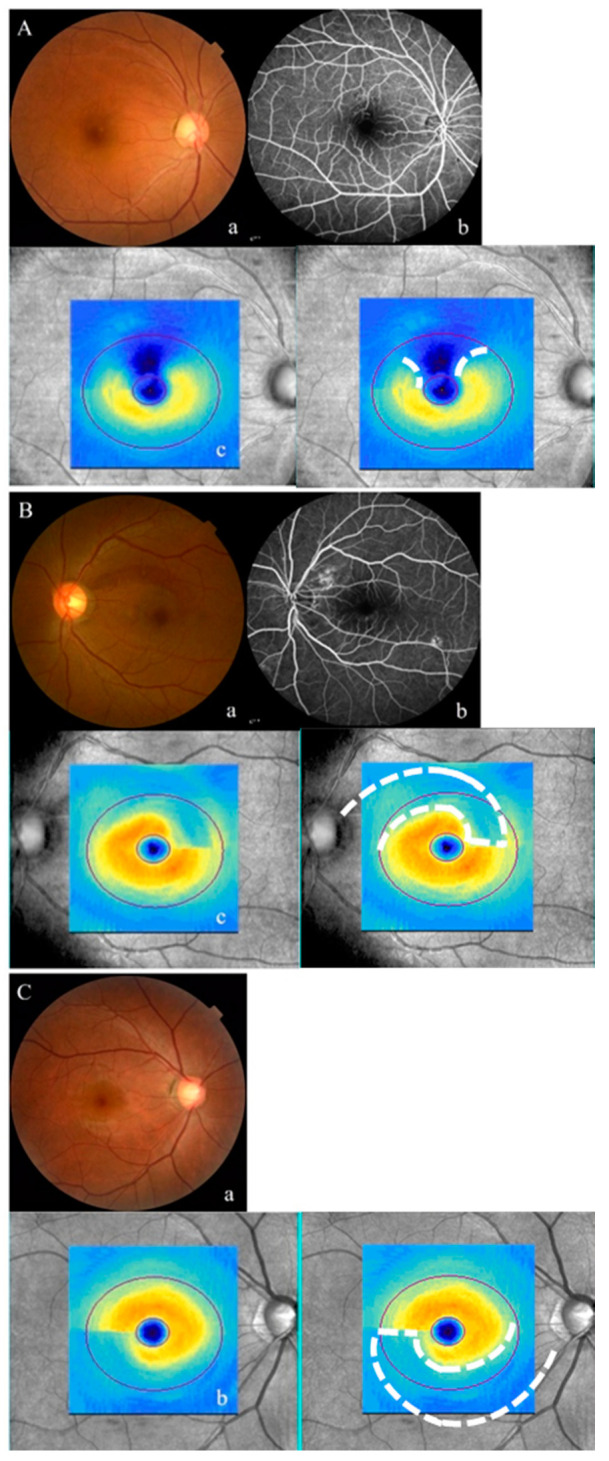
Representative cases from each patient group: patients (**A**) and (**B**) have retinal vein occlusion (RVO) and patient (**C**) has primary open-angle glaucoma (POAG). In patient (**A**), a fundus photograph (a) shows a superotemporal retinal nerve fiber layer (RNFL) defect. Fluorescein angiography (b) shows superiorly localized abnormal retinal vessels and small fovea non-perfusion. The thickness map of the ganglion cell analysis (GCA) (c) shows non-arcuate and interrupted ganglion cell-inner plexiform layer (GCIPL) thinning with blue/black color in the superior hemisphere. In patient (**B**), a superotemporal RNFL defect (a) and corresponding regularly arcuate GCIPL thinning on the thickness map (c) are shown. Mild leakage of the fluorescein from an abnormal retinal vessel localized in the superotemporal peripapillary area is shown (b). In patient (**C**), an inferotemporal RNFL defect (a) and corresponding regularly arcuate GCIPL thinning (b) are shown.

First, the GCA thickness maps of the representative patients in both RVO (Figure 1A) and POAG (Figure 1C) were shown to two masked general ophthalmologists (HML, KSP), with simple explanations of the one distinguishing characteristic of the two conditions: in RVO, an interrupted and irregular topographic change and in POAG, arcuate and smooth topographic change. Second, the two examiners were presented with all GCA thickness maps without any other information (no other SD-OCT images, fundus photographs, or visual field test results). Thus, the examiners were forced to make a decision regarding a diagnosis of RVO or POAG based solely on the GCIPL thinning pattern on the GCA thickness map. The two examiners worked independently of each other.

### 2.6. Statistical Analyses

Inter-observer agreement for a decision between RVO and POAG based on the GCA thickness map was assessed using kappa statistics [19]. A quantitative comparison of the thickness parameters of RNFL, GCIPL, and overall macular thickness between RVO and POAG was conducted with analysis of covariance (ANCOVA). Age, sex, average RNFL thickness, and the signal strength of SD-OCT scans were adjusted. Additionally, we conducted subgroup analyses for RVO patients with the same thickness parameters of RNFL, GCIPL, and overall macular thickness with ANCOVA between patients with an irregular thickness map (diagnosed as RVO based on the GCA thickness map) and patients with a regular thickness map (misdiagnosed as POAG based on the GCA thickness map). Statistical analyses were performed using SPSS version 18.0 (SPSS, Inc., Chicago, IL, USA). A *p*-value < 0.05 was considered statistically significant.

## 3. Results

Thirty-seven patients with RVO and 74 patients with POAG were enrolled. Table 1 lists the demographics of the two disease groups. In RVO, the ‘damaged hemisphere’ appeared in the superior hemisphere in 24 patients (64.9%) and in the inferior hemisphere in 13 patients (35.1%). In POAG, the ‘damaged hemisphere’ was superior in 16 patients (21.6%) and inferior in 58 patients (78.4%, *p* < 0.001). The average RNFL thicknesses of RVO and POAG groups were 84.81 ± 9.45 and 83.13 ± 8.94 μm (*p* = 0.376), respectively. The average GCIPL thicknesses of RVO and POAG groups were 73.43 ± 9.91 and 73.17 ± 6.40 μm (*p* = 0.963), respectively. No differences were observed in the signal strengths of optic disc cube scans or macular cube scans in either of the two groups (*p* = 0.675 and 0.237, respectively).

Table 2 lists the results of the comparison of RNFL thickness and GCIPL thickness between the two disease groups. Among the quadrant RNFL thickness, only temporal thickness was significantly thinner in the RVO group compared with the POAG group (64.94 ± 8.39 versus 70.30 ± 8.35 μm, respectively, *p* = 0.003). Minimum GCIPL thickness was significantly thinner in patients with RVO than those with POAG (51.50 ± 13.70 vs. 60.35 ± 13.60 μm, *p* = 0.002). Sectoral GCIPL thickness did not differ significantly between the two groups.

Table 3 lists the comparison results of macular thickness between RVO and POAG patients. Central macular thickness did not differ significantly (RVO, 242.84 ± 20.75 vs. POAG, 249.57 ± 20.59 μm, respectively, *p* = 0.132). Inner circle macular thickness in individual quadrants did not differ between the two groups, whereas the inferior and temporal quadrants of the outer circle macular thickness were significantly thicker in the RVO group (*p* = 0.003 and 0.002, respectively).

**Figure 2:**
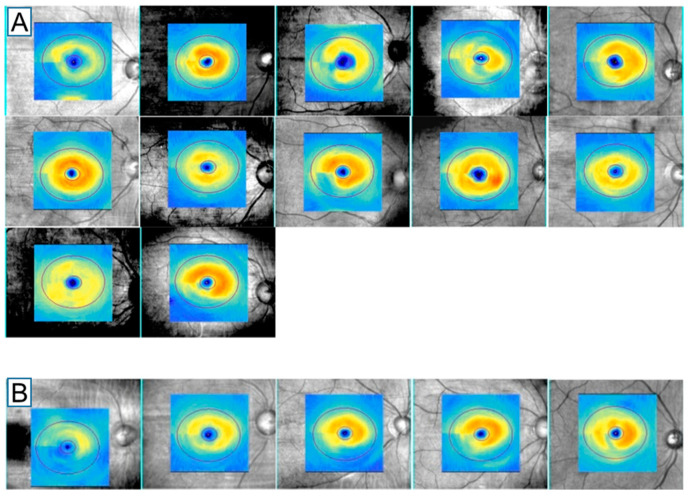
Thickness maps of the ganglion cell analysis (GCA) of (**A**) 12 retinal vein occlusion (RVO) patients diagnosed as primary open-angle glaucoma (POAG) by one or two examiners and (**B**) 5 POAG patients diagnosed as RVO by one or two examiners.

The readings of the thickness maps from GCA from the two independent examiners are shown in Supplementary Figure S1 and Figure 2A for RVO patients and in Supplementary Figure S2 and Figure 2B for POAG patients. For convenience, the thickness map acquired in the left eye was reversed into the right; in patients with a superiorly ‘damaged hemisphere,’ the map was inverted upside down. The two examiners made the same reading of RVO in 25 of 37 patients with RVO (Supplementary Figure S1). The other 12 patients had a reading as POAG from at least one of the two examiners (Figure 2A). In 74 patients with POAG, 69 patients had a consistent reading of POAG (Supplementary Figure S2), but five patients had a reading as RVO from at least one of the two examiners (Figure 2B). Strength of inter-observer agreement was good, at 75.7%, with a kappa value of 0.765 (95% confidence interval, 0.634–0.896, *p* < 0.001). The diagnostic specificity of RVO using the GCA thickness map from POAG was 93.2% (69 patients of Figure 2A/(69 patients of Figure 2A + 5 patients of Figure 2B)) and sensitivity was 67.6% (25 patients of Figure 1A/(25 patients of Figure 1A + 12 patients of Figure 1B)). Diagnostic accuracy was 80.4%.

Table 4 lists the results of the subgroup analysis; patients with RVO were divided into two groups based on consistency from the two examiners of the GCA thickness map readings (Figure 2A vs. Supplementary Figure S1). Patients with inconsistent readings of RVO (regular thickness map) were significantly older than patients with consistent readings (irregular thickness map) (68.42 ± 11.90 years vs. 61.04 ± 8.64 years, respectively, *p* = 0.019). The average RNFL and GCIPL thicknesses of patients with an irregular thickness map did differ significantly from those in patients with a regular thickness map (85.20 ± 8.92 vs. 84.00 ± 10.85 μm and 71.60 ± 10.86 vs. 77.25 ± 6.37 μm, *p* = 0.723 and 0.105, respectively).

Table 5 lists the comparison results of RNFL thickness, GCIPL thickness, and macular thickness between the two sub-groups of RVO patients. The superior quadrant RNFL thickness in patients with an irregular GCA thickness map was thicker than that in patients with a regular GCA thickness map (*p* = 0.018). Minimum, inferior, and inferotemporal GCIPL thicknesses in patients with an irregular thickness map were thinner than those in patients with a regular map (*p* < 0.001, 0.014, and 0.023, respectively). The inferior quadrant of the inner circle macular thickness was thinner in patients with an irregular thickness map than in those with a regular thickness map (*p* = 0.029), and the superior quadrant of outer circle macular thickness was thicker in patients with an irregular thickness map than in those with a regular thickness map (*p* = 0.029).

## 4. Discussion

This study investigated the usefulness of the GCA thickness map for differentiation of RVO from POAG. Based on consistent readings from independent examiners, the diagnostic specificity of RVO using the GCA thickness map was relatively good (93.2%) and the diagnostic accuracy was 80.4%. In a quantitative comparison, temporal RNFL thickness, minimum GCIPL thickness, and inferior and temporal outer circle macular thicknesses were significantly thinner in RVO patients than in POAG patients.

The association between RVO and glaucoma has been reported in many studies [20,21,22,23,24,25,26,27,28]. The prevalence of glaucoma is significantly higher in patients with RVO than in the general population. Also, glaucoma in the fellow eye was significantly more common in patients with RVO than in controls [26,27]. Recently, Yin et al. [23] reported the results of a meta-analysis of the association of glaucoma with risk of RVO; relevant studies were identified by searching in PubMed, EMBASE, and Cochrane up to February 2018. This meta-analysis revealed that glaucoma is associated with the risk of RVO. Although there has been some controversy [29], many previous studies have indicated common pathological mechanisms and risk factors underlying the development of RVO and glaucoma [24,25,26,27,28]. Meanwhile, it is clear that glaucoma and RVO have different clinical courses, as a progressive disease and as a non-progressive disease, respectively, that require completely different therapeutic strategies. In patients with glaucoma, lifelong treatment with intraocular pressure-lowering medication is necessary to prevent progression. If RVO patients have no acute symptoms (i.e., a reduction in visual acuity) and no remaining retinal vascular abnormality, they may be misdiagnosed as having glaucoma and thereby subjected to unnecessary lifelong treatment.

To date, no study has evaluated the usefulness of GCA of SD-OCT for differentiating these two diseases. We expected that RNFL thickness values obtained using SD-OCT would be insufficient with regard to differentiating between the two conditions, as RNFL thinning occurs in not only glaucoma but also RVO [9]. The GCA algorithm in macular cube scans acquired using a Cirrus HD OCT system offers a thickness map based on the entire set of cube data and a sector-based deviation map and thickness values; the sector-based deviation map and thickness values are depicted with stepwise yellow and red color codes obtained from a comparison of the GCIPL thickness with normative data that is abnormally thin at the 5% and 1% levels, respectively. The GCA thickness map provides overall topographic data, regardless of differences from normative data. According to previous studies, the GCA thickness map has good diagnostic sensitivity in early glaucoma, in which the hemifield difference across the temporal raphe is used as diagnostic criteria of glaucoma [10,11,12]. Although it has not been used for diagnostic criteria, a smooth and arcuate GCIPL thinning pattern in a GCA thickness map is also characteristic of glaucoma (Figure 1C) [10,11,12]. We expect that an irregular and non-arcuate GCIPL thinning pattern in a GCA thickness map can implicate RVO (Figure 1A) other than glaucoma. To evaluate the usefulness of an irregular GCIPL thinning pattern, we enrolled branch RVO and hemi-central RVO possibly having hemifield differences across the temporal raphe similar with glaucoma. According to our study results, the irregularity of the thickness map yielded relatively good specificity (93.2%), but poor sensitivity (67.6%) in differentiating RVO from glaucoma. In other words, if an irregular thinning pattern is observed in the GCA thickness map, the possibility of another disease, such as RVO, should be considered, despite evidence of RNFL thinning and corresponding visual field defects suspicious of glaucoma. However, RVO cannot be excluded based only on a regular GCIPL thinning pattern (Figure 1B).

Comparison of the peripapillary RNFL thickness revealed that the temporal RNFL thickness in RVO was thinner than in glaucoma. A possible reason for this is that in this study, we enrolled patients with glaucoma matched to RVO with average RNFL thickness; the papillomacular bundle of retinal nerve fiber located in the temporal area is commonly saved until the terminal stage in glaucoma. In a comparison of macular cube scans, although no difference was observed in inferior and inferotemporal sector GCIPL thicknesses, the inferior and temporal outer circle overall macular thickness was significantly thicker in RVO patients compared with POAG patients. These results may be explained by two possible situations. First, in POAG patients, the GCIPL thickness between the outside of the elliptical annulus (dimensions, 4 mm × 4.8 mm) in GCA and inside of the outer circle margin (diameter, 6 mm) in macular cube scans was thinner than in RVO patients. Second, in RVO, subclinical macular edema without cystic space remained. The GCIPL thinning pattern in GCA thickness maps can be affected by not only abrupt GCIPL thinning but also by the remaining focal macular edema. The minimum GCIPL thickness in RVO was significantly thinner than in POAG. This seems to be associated with an irregular GCIPL thickness map due to abrupt GCIPL thinning. In other words, GCIPL thickness in the most damaged location is expected to be thinner in RVO than in POAG matched with the average RNFL thickness.

We conducted a subgroup analysis of RVO patients in an attempt to determine the factors related to the diagnostic sensitivity of the GCA thickness map. No demographic differences were observed between groups with an irregular versus a regular GCA thickness map, with the exception of age. No differences in average RNFL or GCIPL thicknesses were observed, whereas inferior thickness values and minimum GCIPL thickness were thinner, and superior thickness values were thicker, in the irregular GCIPL thickness group. According to these results, RVO with more localized and concentrated damage can facilitate a more irregular GCA thickness map. Our speculation is based on the fact that unlike glaucoma, RNFL thinning and GCIPL thinning in RVO is secondary to retinal vascular occlusion. Retinal nerve fiber and ganglion cells are located within a uniform and arcuate pathway and do not cross each other’s path [30,31]. In contrast, the retinal vascular pathway does not arcuate uniformly; a major branch retinal vein may appear arcuate, but secondary and tertiary branches and capillaries develop into a network rather than an arc [32,33]. Manabe et al. reported parafoveal capillary nonperfusion frequently in patients with completely resolved branch RVO (25 of 27 eyes, 92.6%) [33].

In conclusion, we evaluated the usefulness of GCA thickness maps for differentiating RVO from POAG. Based on our study results, GCA thickness maps provide a simple and convenient way to differentiate a diagnosis of RVO from one of POAG. If the GCA thickness map shows an abrupt and/or non-arcuate GCIPL thinning pattern in patients who have RNFL thinning suspicious of glaucoma, further evaluation such as angiography is needed to confirm RVO versus glaucoma.

## Figures and Tables

**Table 1:** Demographics of patients with retinal vein occlusion and primary open-angle glaucoma.

	RVO (*n* = 37)	POAG (*n* = 74)	*p*-Value *
Age (years)	63.43 ± 10.26	61.81 ± 12.53	0.476
Sex (male/female)	13/24	34/40	0.277
BCVA (decimal)	0.94 ± 0.16	1.01 ± 0.24	0.109
Axial length (mm)	23.86 ± 0.91	23.59 ± 1.10	0.391
Intraocular pressure (mmHg)	14.97 ± 2.67	15.97 ± 2.82	0.186
Damaged hemisphere (%) †			<0.001 ‡
Superior	24 (64.86)	16 (21.62)	
Inferior	13 (35.14)	58 (78.38)	
Signal strength of optic disc scan	9.30 ± 0.66	9.35 ± 0.65	0.675
Average RNFL thickness (μm)	84.81 ± 9.45	83.13 ± 8.94	0.376
Signal strength of macular scan	9.22 ± 0.75	9.00 ± 0.81	0.237
Average GCIPL thickness (μm)	73.43 ± 9.91	73.17 ± 6.40	0.963

RVO = retinal vein occlusion, POAG = primary open-angle glaucoma, BCVA = best-corrected visual acuity, RNFL = retinal nerve fiber layer, GCIPL = ganglion cell-inner plexiform layer. * Independent t-test. † In patients with RVO, a damaged hemisphere was indicated for a hemisphere with vascular occlusion. In POAG, a more damaged hemisphere had a thinner peripapillary RNFL thickness. ‡ Pearson’s chi square test.

**Table 2:** Comparison of peripapillary RNFL and macular GCIPL thickness in patients with retinal vein occlusion and primary open-angle glaucoma.

	RVO (*n* = 37)	POAG (*n* = 74)	*p*-Value *
Quadrant RNFL thickness (μm) †			
Superior ‡	113.06 ± 10.28	109.66 ± 10.22	0.113
Nasal	63.75 ± 7.66	65.18 ± 7.62	0.371
Inferior ‡	93.06 ± 11.98	89.89 ± 11.91	0.205
Temporal	64.94 ± 8.39	70.30 ± 8.35	0.003
Minimum GCIPL thickness (μm) §	51.50 ± 13.70	60.35 ± 13.60	0.002
Sectoral GCIPL thickness (μm) §			
Superotemporal #	79.08 ± 6.06	76.75 ± 6.02	0.068
Superior ‡	79.96 ± 5.07	79.36 ± 5.03	0.571
Superonasal ¶	82.27 ± 6.36	81.89 ± 6.31	0.772
Inferonasal ¶	74.45 ± 11.08	74.24 ± 11.00	0.929
Inferior ‡	60.71 ± 13.06	64.78 ± 12.96	0.137
Inferotemporal #	61.84 ± 11.28	62.07 ± 11.20	0.922

RVO = retinal vein occlusion, POAG = primary open-angle glaucoma, RNFL = retinal nerve fiber layer, GCIPL = ganglion cell-inner plexiform layer. * General linear model with analysis of covariance. † Age, sex, average RNFL thickness, and signal strength of optic disc scan adjusted mean ± SD. § Age, sex, average RNFL thickness, and signal strength of macular scan adjusted mean ± SD. ‡, ¶, # In patients with a ‘damaged hemisphere’ in the superior hemisphere, the thickness parameters of this hemisphere were converted upside down: superior interchanged with inferior, superotemporal with inferotemporal, and superonasal with inferonasal. As a result, the inferior hemisphere was the ‘damaged hemisphere’ in all patients.

**Table 3:** Comparison of the overall macular thickness in patients with retinal vein occlusion and primary open-angle glaucoma.

	RVO (*n* = 37)	POAG (*n* = 74)	*p*-Value †
Central macular thickness (μm)	242.84 ± 20.75	249.57 ± 20.59	0.132
Inner circle macular thickness (μm)			
Superior *	316.96 ± 14.41	319.76 ± 14.30	0.352
Nasal	314.88 ± 19.39	319.91 ± 19.25	0.215
Inferior *	288.55 ± 31.20	298.82 ± 30.97	0.117
Temporal	297.40 ± 17.95	297.79 ± 17.82	0.918
Outer circle macular thickness (μm)			
Superior *	273.37 ± 13.69	271.41 ± 13.58	0.492
Nasal	293.90 ± 17.69	289.00 ± 17.56	0.185
Inferior *	260.88 ± 25.36	243.42 ± 25.17	0.001
Temporal	259.20 ± 13.65	249.28 ± 13.54	0.001

RVO = retinal vein occlusion, POAG = primary open-angle glaucoma. Age, sex, average peripapillary RNFL thickness, and signal strength of macular scan adjusted mean ± SD. * In patients with a ‘damaged hemisphere’ in the superior hemisphere, thickness parameters in this hemisphere were converted upside down: superior interchanged with inferior. As a result, the inferior hemisphere was the ‘damaged hemisphere’ in all patients. † Independent t-test.

**Table 4:** Demographics of patients with retinal vein occlusion.

	Irregular GCA Thickness Map (*n* = 25)	Regular GCA Thickness Map (*n* = 12)	*p*-Value *
Age (years)	61.04 ± 8.64	68.42 ± 11.90	0.019
Sex (male/female)	10/15	3/9	0.476 †
BCVA (decimal)	0.94 ± 0.12	0.93 ± 0.23	0.761
Axial length (mm)	24.09 ± 0.80	23.43 ± 0.97	0.094
Intraocular pressure (mmHg)	15.20 ± 2.25	16.58 ± 2.84	0.190
Damaged hemisphere ‡			0.149 †
Superior	14	10	
Inferior	11	2	
Signal strength of optic disc scan	9.23 ± 0.67	9.38 ± 0.78	0.394
Average RNFL thickness (μm)	85.20 ± 8.92	84.00 ± 10.85	0.723
Signal strength of macular scan	9.31 ± 0.71	9.01 ± 0.72	0.209
Average GCIPL thickness (μm)	71.60 ± 10.86	77.25 ± 6.37	0.105

RVO = retinal vein occlusion, GCA = ganglion cell analysis, BCVA = best-corrected visual acuity, RNFL = retinal nerve fiber layer, GCIPL = ganglion cell-inner plexiform layer. * Independent t-test. † Fisher’s exact test. ‡ Damaged hemisphere means a hemisphere with RVO.

**Table 5:** Peripapillary RNFL, macular GCIPL, and macular thickness in patients with retinal vein occlusion.

	Irregular GCA Thickness Map (*n* = 25)	Regular GCA Thickness Map (*n* = 12)	*p* Value
Quadrant RNFL thickness (μm) *			
Superior ‡	117.54 ± 9.39	108.87 ± 9.67	0.018
Nasal	65.39 ± 3.35	62.77 ± 7.57	0.343
Inferior ‡	92.38 ± 10.78	98.97 ± 11.10	0.109
Temporal	63.60 ± 7.88	68.83 ± 8.11	0.084
Minimum GCIPL thickness (μm) †	45.31 ± 14.35	67.35 ± 15.16	<0.001
Sectoral GCIPL thickness (μm) †			
Superior ‡	80.81 ± 5.91	77.31 ± 6.24	0.138
Superonasal §	83.71 ± 8.74	78.86 ± 9.23	0.164
Inferonasal §	72.87 ± 14.70	80.03 ± 15.53	0.219
Inferior ‡	56.11 ± 16.36	72.77 ± 17.29	0.014
Inferotemporal ¶	58.69 ± 13.14	70.90 ± 13.88	0.023
Superotemporal ¶	79.03 ± 8.02	79.44 ± 8.47	0.894
Central macular thickness (μm) †	240.40 ± 25.14	247.00 ± 26.56	0.504
Inner circle macular thickness (μm) †			
Superior ‡,	318.47 ± 14.89	311.44 ± 15.73	0.234
Nasal	316.39 ± 24.79	312.01 ± 26.19	0.652
Inferior ‡	279.09 ± 34.51	309.73 ± 36.46	0.029
Temporal	295.68 ± 22.96	302.17 ± 24.25	0.472
Outer circle macular thickness (μm) †			
Superior ‡	278.62 ± 12.88	261.46 ± 13.61	0.002
Nasal	295.28 ± 20.09	291.75 ± 21.23	0.654
Inferior ‡	258.85 ± 34.92	270.81 ± 36.89	0.385
Temporal	260.56 ± 16.87	258.09 ± 17.82	0.709

RNFL = retinal nerve fiber layer, GCIPL = ganglion cell-inner plexiform layer, RVO = retinal vein occlusion, GCA = ganglion cell analysis. * Age, sex, average RNFL thickness, and signal strength of optic disc scan adjusted mean ± SD. † Age, sex, average RNFL thickness, and signal strength of macular scan adjusted mean ± SD. ‡, §, ¶ In patients with superior RVO, RNFL thickness, GCIPL thickness, and macular thickness in the superior hemisphere are converted upside down: superior interchanged with inferior, superotemporal with inferotemporal, and superonasal with inferonasal. As a result, the inferior hemisphere was the ‘damaged hemisphere’ in all patients.

## Supplementary Materials

The following are available online at https://www.mdpi.com/2077-0383/9/10/3294/s1, Figure S1: Thickness maps of the ganglion cell analysis of retinal vein occlusion, Figure S2: Thickness maps of the ganglion cell analysis of primary open-angle glaucoma.
