# Supplementary material for: Using the Thickness Map from Macular Ganglion Cell Analysis to Differentiate Retinal Vein Occlusion from Glaucoma
Source: J Clin Med. 2020 Oct 14;9(10):3294. doi: 10.3390/jcm9103294 (PMC7602489; doi:10.3390/jcm9103294)
Supplement: Supplementary file 1 [file jcm-09-03294-s001.pdf]

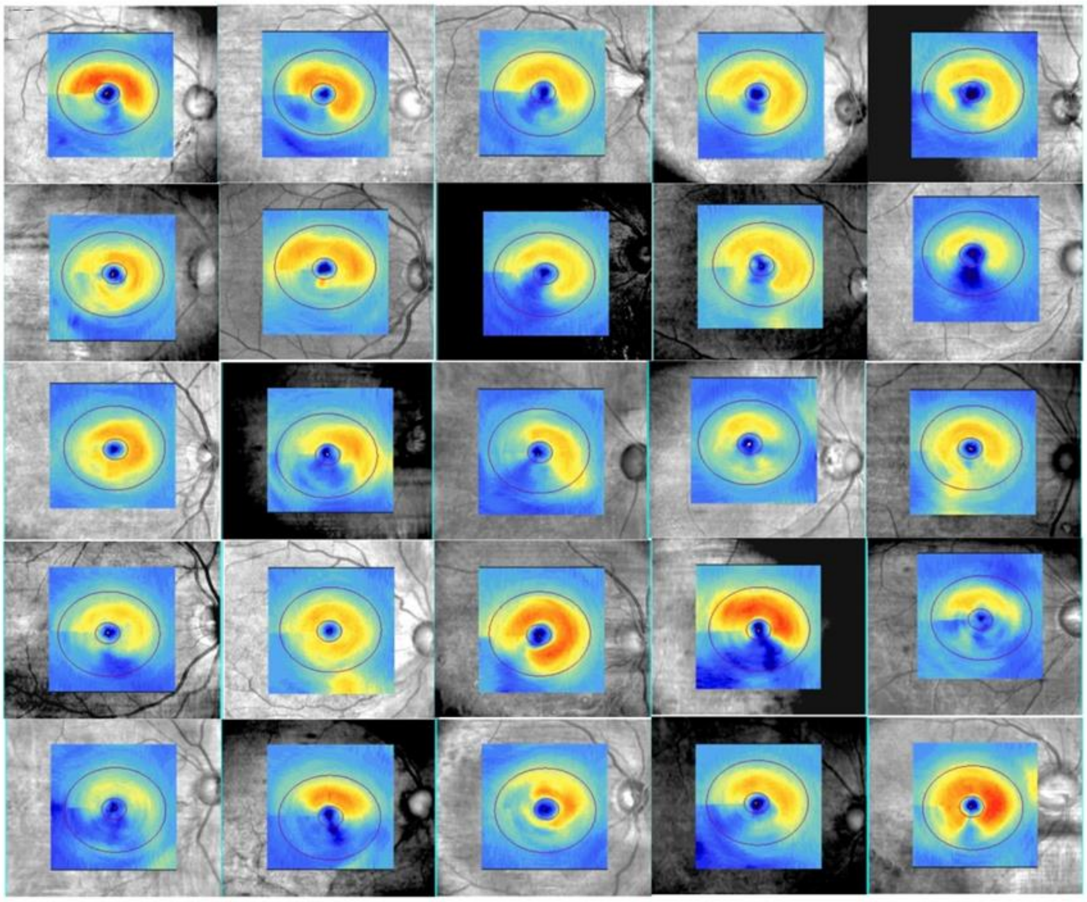

Supplement 1. Thickness maps of the ganglion cell analysis (GCA) of 25 retinal vein occlusion (RVO) patients diagnosed consistently as RVO by two independent examiners.

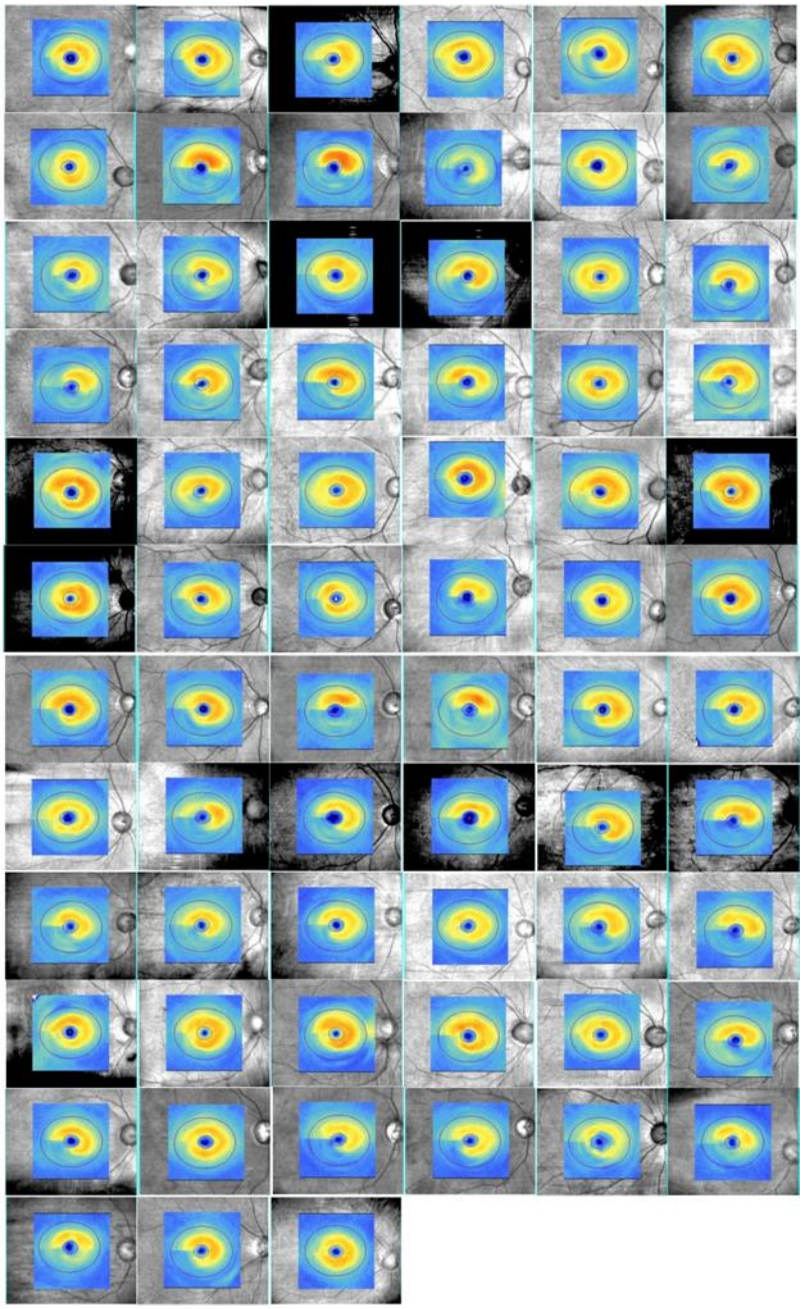

Supplement 2. Thickness maps of the ganglion cell analysis (GCA) of 69 primary open-angle glaucoma (POAG) patients diagnosed consistently as POAG by two independent examiners.
